# Effect of Combined Addition of CeLa and GdY on Microstructure and Mechanical Properties of As-Cast Al-Cu-Mn Alloys

**DOI:** 10.3390/ma16237332

**Published:** 2023-11-25

**Authors:** Haiyang Zhang, Mingdong Wu, Zeyu Li, Daihong Xiao, Yang Huang, Lanping Huang, Wensheng Liu

**Affiliations:** National Key Laboratory of Science and Technology on High-Strength Structural Materials, Central South University, Changsha 410083, China; 223311047@csu.edu.cn (H.Z.); mmingshiddong@csu.edu.cn (M.W.);

**Keywords:** Al-Cu-Mn casting alloy, rare earth micro-alloying, microstructure, mechanical properties

## Abstract

In this study, the effects of the combined addition of CeLa and GdY on the microstructure and mechanical properties of as-cast Al-4Cu-1Mn alloys were investigated by X-ray diffraction (XRD), scanning electron microscopy (SEM), transmission electron microscopy (TEM), and tensile testing. The results show that the minor addition of CeLa and GdY leads to a refinement of grain size. The addition of CeLa results in the formation of supersaturated vacancies in the Al matrix, whereas the addition of GdY leads to a decrease in the precipitation temperature of the Al_2_Cu phase. The combined CeLa and GdY additions can significantly increase ultimate tensile strength (UTS) while losing only a small amount of elongation (EL). Compared with the unmodified alloy, the grain size and SDAS of the alloy (0.2 wt.% CeLa + 0.1 wt.% GdY) were diminished by 67.2% and 58.7%, respectively, while maximum hardness and UTS rose by 31.2% and 36.9%, respectively.

## 1. Introduction

Al-Cu-Mn alloys have been extensively used in military and automotive applications due to their high strength, good corrosion resistance, and excellent fatigue properties [1,2]. In the preparation of materials, we constantly strive for high overall performance. Unlike deformable aluminum alloys, the properties of as-cast Al-Cu-Mn alloys are highly dependent on the melting and casting processes. During the casting process, casting defects (such as hot cracks and shrinkage cavities) are inevitable to form, which limits their widespread application [3,4]. Therefore, improving their castability is particularly important to improve the microstructure and mechanical properties of as-cast Al-Cu-Mn alloys.

Adding small amounts of rare earth (RE) such as Ce [5,6,7], La [8,9,10], Sm [11,12,13], Sc [14,15], Gd [16,17,18], and Er [19,20,21] has been proven to play a good role in the purification and densification of aluminum alloy melt, reducing casting defects and making the microstructure more dense [22,23,24]. At the same time, the addition of RE can refine the grain size of the as-cast alloy by promoting composition overcooling [25]. Yao et al. found that adding 0.3 wt.% La had a significant refining effect on Al-Cu alloy grains due to their segregation and collection in the solidification front [8]. The measured average grain sizes are 0.155 and 0.035 mm for the Al-Cu alloys without La and with La, respectively. The addition of RE can also promote the formation of RE-rich phases and improve the comprehensive properties of aluminum alloys [26]. For example, Amer et al. discovered the existence of the Al_8_Cu_4_Gd phase in Al-Cu alloys when 0.25 wt.% Gd was added [16]. These phases improved the mechanical properties of Al-Cu alloys. Guo et al. found that the strength and hardness of the alloys with the addition of 0.1 wt.% Y were increased due to the existence of the Al_8_Cu_4_Y phase [27]. After T6 heat treatment, the alloy containing 0.1 wt.% Y (181 HV) exhibits a 48% increase in hardness compared to the unalloyed alloy (122 HV). The individual RE (Ce [28], La [29], Gd [30] and Y [27]) can all modify Al-Cu-Mn alloys to enhance their overall performance. However, excessive addition of individual RE can lead to coarse grains and excessive intermetallic compounds, which have a detrimental effect on the mechanical properties.

The literature has reported the effect of the combined addition of CeLa [31,32], LaY [33], CeY [34], LaPr [35], and LaSc [36] on the microstructure and properties of Al-Cu alloys. The results show that binary combined additions of Re have a significant effect on the grain refinement of the alloy. Liu et al. [37] studied the impact of 0.3 wt.% LaCe on the solidification process of Al-Cu alloys. Their findings indicated that CeLa could increase melt subcooling by inhibiting the nucleation of α-Al on large heterogeneous particles, resulting in grain refinement. Shuai et al. found that when 0.6 wt.% LaY was added to the Al-Si-Cu alloy, the SDAS of α-Al decreased to 77.56 µm, which is 75.11% lower than Al-Si-Cu [33]. Furthermore, binary combined additions of Re demonstrated better performances than single additions of rare earth elements. Wang et al. found that the simultaneous addition of CeY had a better grain refinement effect than the addition of Ce [34] alone. The average grain size of the alloy was 150.04 ± 5 µm when Ce alone was added, but was reduced to 90.23 ± 5 µm with the addition of CeY. Song et al. added La and LaSc to Al-Cu alloys [36]. The results showed that the grain size of Al-Cu-La-Sc alloy (69.25 µm) was smaller than that of Al-Cu-La alloy (118.53 µm). However, binary combined additions of RE above a certain amount can also have an adverse effect on the mechanical properties of the alloy. Du et al. [38] investigated the microstructure and mechanical properties of Al-Cu-Mn alloys with different CeLa additions. The results showed that the addition of 0.25 wt.% CeLa improved the room-temperature mechanical properties of the alloy, but the addition of 0.5 wt.% CeLa promoted the formation of coarse Al_8_Cu_4_Ce and Al_6_Cu_6_La phases, resulting in a decrease in mechanical properties.

Based on the above analysis, the multi-compound addition of RE can be used as a potential way to reduce the casting defects of alloys. Furthermore, the multi-compound addition of RE presents the opportunity to achieve highly refined grains and generate a suitable quantity of intermetallic compounds using a lower total amount of RE, resulting in superior mechanical properties. Previous [34,36] literature has reported the effect of LaSc or LaPr in Al-Cu alloys, respectively. Although the RE (Sc or Pr) has a good modifying effect, the RE has a high price and is not suitable for large-scale applications. The storage capacity of RE (Ce, La, Gd, and Y) accounts for the largest proportion of all rare earth elements, and their prices are 80–100 times lower than Sc. Moreover, as the by-product of Sc, increasing the utilization rate of RE (Ce, La, Gd, and Y) can achieve the purpose of saving the earth’s resources and reducing environmental pollution, just like biodegradable materials [39]. Therefore, it would be an interesting research direction to study the effect of the combined addition of Ce, La, Gd, and Y on the microstructure and mechanical properties of Al-Cu alloys. However, little research has investigated the effect of the combined addition of CeLa and GdY on Al-Cu-Mn alloys.

In this study, the effects of combined additions of CeLa and GdY on the microstructure and mechanical properties of cast Al-Cu-Mn alloys at both room temperature and T6 heat treatment and the relevant mechanisms are clarified. The findings in this study can assist in the development of modifiers and broaden the application of Al-Cu-Mn alloys.

## 2. Materials and Methods

The Al-4Cu-1Mn (wt.%)-based alloy was obtained by melting pure Al (purity 99.99%), Al-Cu, and Al-Mn intermediate alloys in the resistance melting furnace. The Al-4Cu-1Mn alloy was completely melted at 780 °C and then held for 10 min, and predetermined amounts of Al-CeLa and Al-GdY master alloys were wrapped in aluminum foil and added to the molten liquid. The melt was held at 760 °C for 20 min, and when the intermediate alloy was completely melted, the whole melt was refined with C_2_Cl_6_. Then, the melt was stirred briefly and kept warm for 10 min. It was then poured into an uncovered cylindrical steel mold with a dimension of φ120 × 200 mm (preheated at 250 °C). Finally, the Al-4Cu-1Mn alloy ingots were obtained with composite additions of CeLa and GdY in various compositions. The alloys were subjected to Inductively Coupled Plasma Atomic Emission Spectrometry (ICP-AES) analysis to determine their chemical composition, and the results are shown in Table 1.

**Figure 1:**
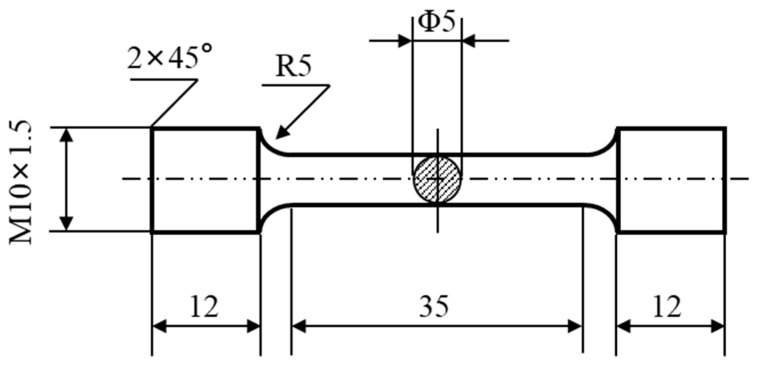
Dimensions of standard tensile specimen of the experimental alloy (Unit: mm).

The physical phase composition of the alloy was determined by X-ray diffractometry (XRD), and the data obtained were analyzed using jade6.5 software. The T6 heat treatment temperature of the as-cast alloy was determined by Differential Scanning Calorimetry (DSC) analysis with a specimen weight of 10 mg and a temperature increase rate of 10 °C/min. The peak aging state was determined by determining the Vickers hardness using a 200HV-5 hardness tester with a load of 5 kg and a duration of 15 s. The average of five points was taken. The room temperature tensile properties of the alloy were measured using the Instron 3369 testing machine at a loading rate of 2.0 mm/min after the alloy was subjected to a T6 heat treatment (solid solution treatment at 535 °C for 16 h and aging at 180 °C for 6 h). The dimensions of the standard tensile specimen are shown in Figure 1. Ultimate Tensile Strength (UTS), Yield Strength (YS), and Elongation (EL) are determined based on the average of three tests. The micro-morphology was characterized by Optical Microscope (OM), Scanning Electron Microscopy (SEM) equipped with electron backscatter diffraction (EBSD), and Transmission Electron Microscopy (TEM) equipped with energy dispersive spectroscopy (EDS). Aztec Crystal and Image Pro Plus 6.0 software were used to determine the specimen grain size and secondary dendrite arm spacing (SDAS). After the tensile test, the fracture was observed with a scanning electron microscope to determine the possible fracture mechanism of the alloy.

## 3. Results

### 3.1. Microstructure

**Figure 2:**
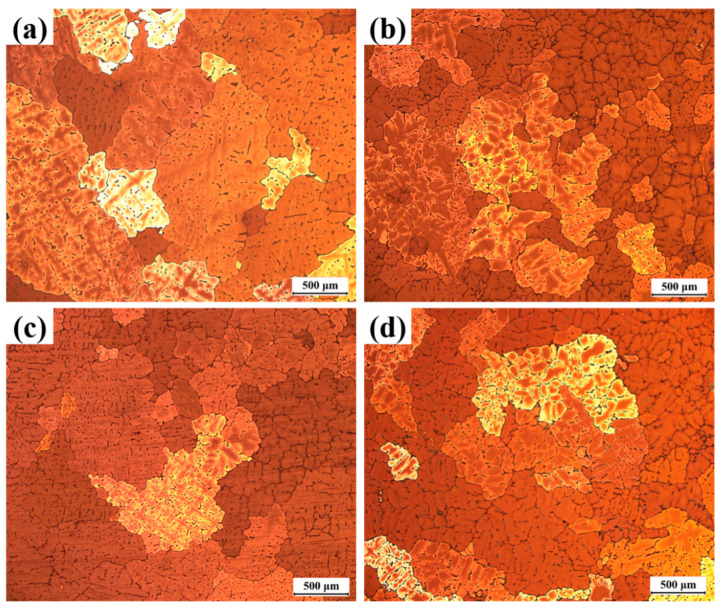
Optical macrographs of as-cast Al-4Cu-1Mn alloys (**a**) Alloy 1, (**b**) Alloy 2 (0.2 CeLa), (**c**) Alloy 3 (0.1 GdY), and (**d**) Alloy 4 (0.2 CeLa + 0.1 GdY).

Figure 2 shows the grain structure corresponding to alloys with different rare earth contents. Compared with the coarse dendrites in the unalloyed Al-4Cu-1Mn alloy (Figure 2a), there was a significant decrease in the coarse α-Al dendrites and an increase in the fine isometric α-Al dendrites in the alloys with the addition of CeLa and GdY, respectively. The grains in the alloys containing CeLa and GdY are predominantly made up of fine equiaxial α-Al dendrites. In addition, it can be found that there are some defects distributed at grain boundaries in the alloy without RE addition (Figure 2a). After the addition of RE, the number of defects in the alloy decreased sharply. This indicates that RE can purify the melt and improve the casting performance of the alloy.

**Figure 3:**
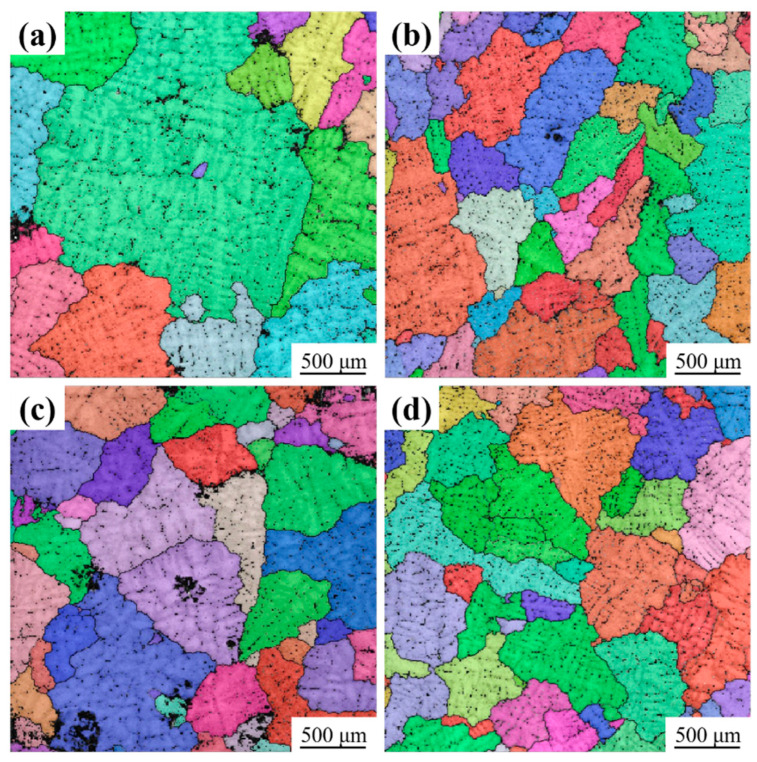
EBSD micrographs of as-cast Al-4Cu-1Mn alloys (**a**) Alloy 1, (**b**) Alloy 2 (0.2 CeLa), (**c**) Alloy 3 (0.1 GdY), and (**d**) Alloy 4 (0.2 CeLa + 0.1 GdY).

**Figure 4:**
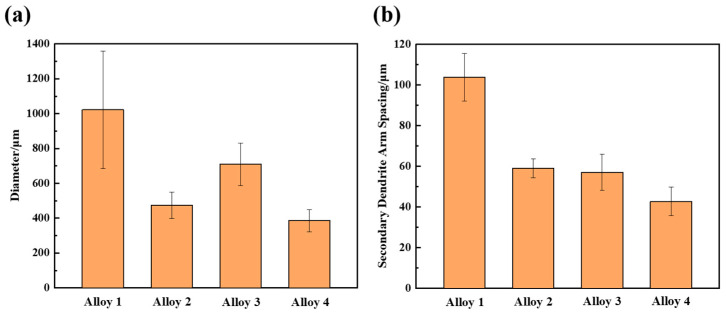
Average grain size (**a**) and SDAS (**b**) of the as-cast Al-4Cu-1Mn alloys.

Figure 3 shows the grain size of the studied alloy. A comparison of grain sizes in alloys with varying RE contents indicates a significant decrease in grain size when RE is added. The grain size and SDAS of the alloy were measured using Aztec Crystal 2.0 and IPP software 6.0, as shown in Figure 4. The results show that the grain size and SDAS of the alloy with CeLa and GdY added are reduced from 1022 ± 337 µm and 103.8 ± 11.7 µm to 386 ± 64 µm and 42.8 ± 11.7 µm, respectively, as compared to the undenatured alloy. However, the grain size and SDAS of the alloy with CeLa alone are 474 ± 76 μm and 59.1 ± 4.7 μm, respectively. On the other hand, the grain size and SDAS of the alloy with GdY addition alone are 710 ± 122 μm and 57.1 ± 8.8 μm, respectively. This suggests that the combined addition of 0.2 CeLa and 0.1 GdY (Figure 3d) may provide better refinement than adding 0.2 CeLa or 0.1 GdY (Figure 3b,c).

**Figure 5:**
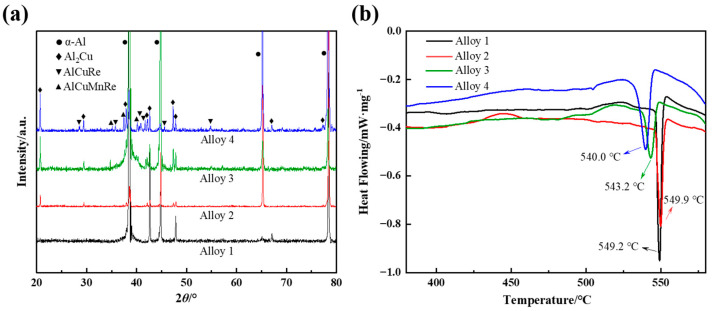
XRD patterns (**a**) and DSC curves (**b**) of the cast Al-4Cu-1Mn alloys.

Figure 5a shows the XRD pattern of the combined addition of different RE-cast alloys. It can be seen that alloy 1 without added RE and alloy 2 with added 0.2 LaCe are mainly composed of α-Al phase and Al_2_Cu phase. However, a small amount of the AlCuMnRE phase is detected in alloy 3 after the addition of GdY. When CeLa + GdY is added, a new phase, the AlCuRE phase, appears in alloy 4, which may be the Al_8_Cu_4_Re phase. Figure 5b shows the DSC curves of the studied alloys, which are selected in the temperature range of 380 to 580 °C. It is observed that a heat absorption peak is observed in this temperature interval, which is the melt heat absorption peak of the Al_2_Cu phase. In addition, the peak heat absorption of the alloy 1 without RE addition is 549.9 °C, which is reduced by the addition of GdY. The combined added CeLa + GdY alloy 4 has the smallest peak heat absorption of 540.0 °C.

**Figure 6:**
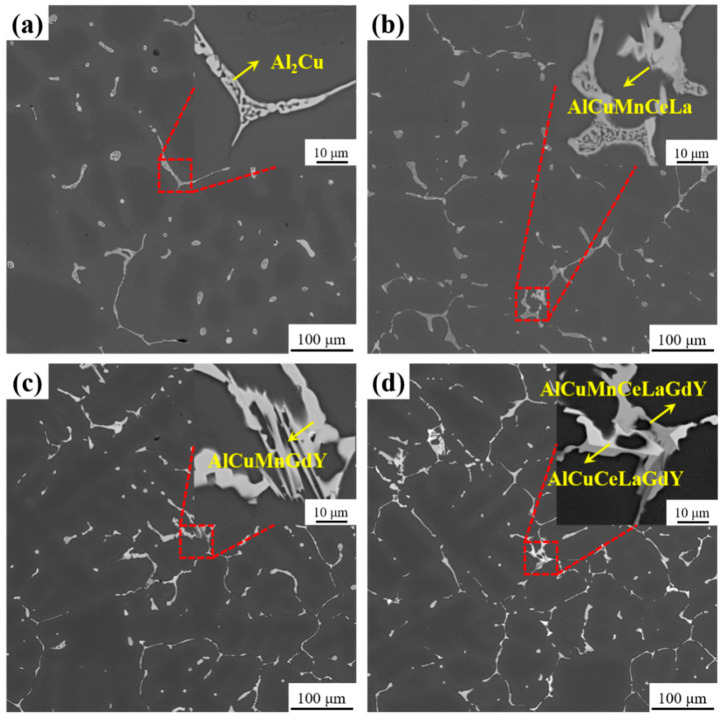
SEM images of Al-4Cu-1Mn alloys as cast (**a**) Alloy 1, (**b**) Alloy 2 (0.2 CeLa), (**c**) Alloy 3 (0.1 GdY), and (**d**) Alloy 4 (0.2 CeLa + 0.1 GdY).

Figure 6 shows the backscattered electron (BSE) images of the cast alloys of Al-4Cu-1Mn with varying RE additions. Most of the intermetallic particles formed in the four alloys are observed to be in spherical and elongated Al_2_Cu phases. The structure of the Al_2_Cu phase in Al-4Cu-1Mn alloys without RE addition is reticulated. It is located almost discontinuously in the interstices of the secondary dendrite arms and at the grain boundaries (Figure 6a). The addition of CeLa decreases the number of circular Al_2_Cu phases and increases the number of elongated Al_2_Cu phases in alloy 2, which are connected to each other to form a network. Further magnification reveals the generation of a gray massive phase around a small amount of Al_2_Cu phase (Figure 6b). After the addition of GdY, as shown in Figure 6c, the volume of the circular Al_2_Cu phase in the alloy 3 decreases significantly, and the number of elongated Al_2_Cu phases becomes finer and connects into a network. Further magnification shows that the Al_2_Cu phase is mostly transformed into gray bulk phases. After the combined addition of CeLa + GdY, the size of the round Al_2_Cu phase in alloy 4 further decreases, and the number of elongated Al_2_Cu phases further increases to form more networks. Further magnification shows that the elongated Al_2_Cu phase mesh structure disappears, and the Al_2_Cu phase is almost completely transformed into a gray massive phase while a new bright bulk phase is generated. Combined with the XRD results, it has been determined that the gray massive phase is AlCuMnRE and the bright massive phase is AlCuRE.

**Figure 7:**
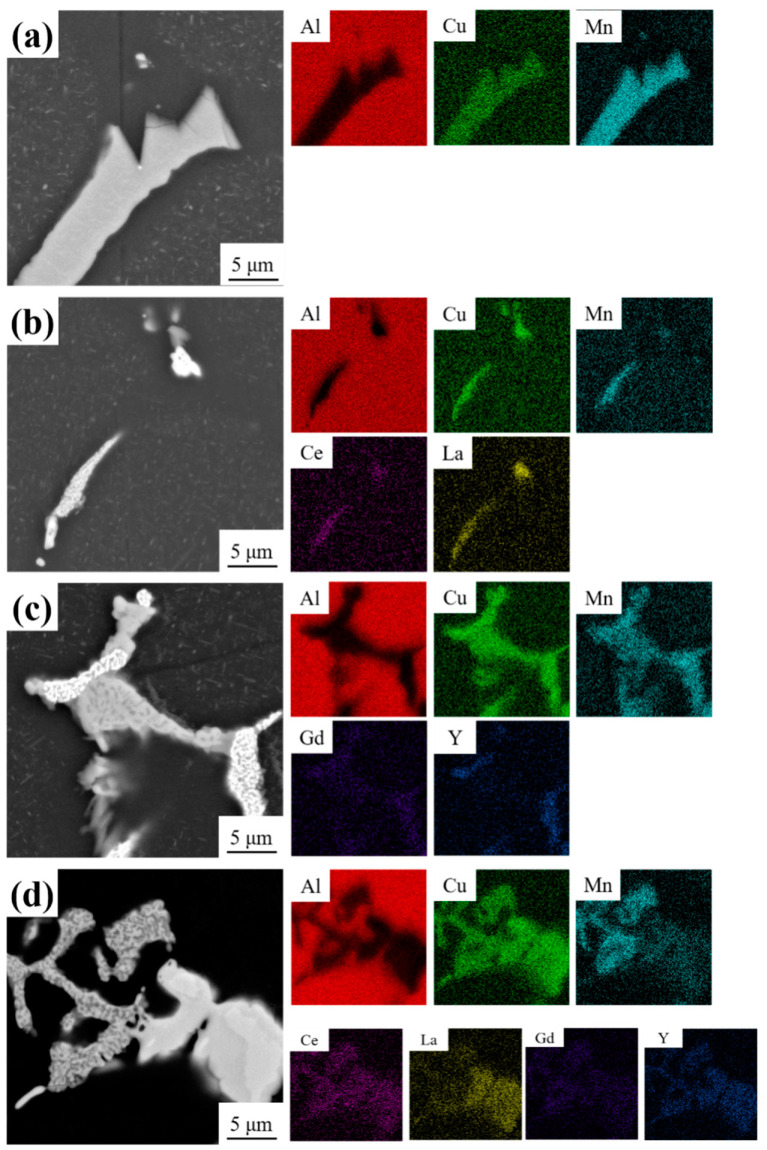
SEM morphologies and EDS of the heat-treated Al-4Cu-1Mn alloys (**a**) Alloy 1, (**b**) Alloy 2 (0.2 CeLa), (**c**) Alloy 3 (0.1 GdY), and (**d**) Alloy 4 (0.2 CeLa + 0.1 GdY).

To further investigate the characteristics of intermetallic compounds in alloys after the addition of RE. Figure 7 displays SEM images and EDS analyses of the intermetallic compounds of Al-4Cu-1Mn alloys with different RE additions after heat treatment. The bulk gray phase in the unalloyed RE alloy consists of Al, Cu, and Mn elements (Figure 7a). With the addition of CeLa, the AlCuMnCeLa phase replaces the grey AlCuMn phase, and a very small amount of bright white AlCuCeLa phase is produced (Figure 7b). With the addition of GdY, some gray phases composed of AlCuMnGd and white phases composed of AlCuMnY are observed (Figure 7c). With the combined addition of CeLa + GdY, the bright white phase of the alloy consists of the elements (Al, Cu, Ce, La, Gd, and Y), and the gray phase consists of the elements (Al, Cu, Mn, Ce, La, Gd, and Y) (Figure 7d). The findings demonstrate that the combined addition of CeLa + GdY changes the morphology of the RE-rich phases and increases their amount.

**Figure 8:**
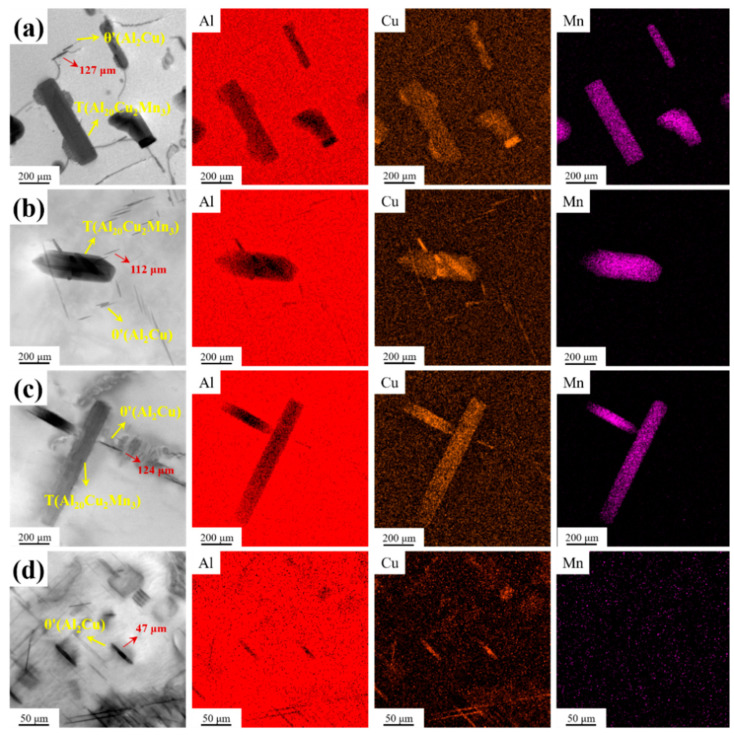
TEM images of the heat-treated Al-4Cu-1Mn alloys (**a**) Alloy 1, (**b**) Alloy 2 (0.2 CeLa), (**c**) Alloy 3 (0.1 GdY), and (**d**) Alloy 4 (0.2 CeLa + 0.1 GdY).

Figure 8 depicts the TEM images of unalloyed and combined CeLa and GdY alloys. The short rod-like particles in Figure 8a–c are in the T(Al_20_Cu_2_Mn_3_) phase, and the needle-like particles in Figure 8d are in the θ′ (Al_2_Cu) phase. It can be seen that alloy 1 without the addition of RE contains more coarse T phases and some needle-like θ′ phases. The quantity of T phases decreases after alloying, especially after the combined addition of CeLa + GdY. The alloy precipitates more θ′ phases with a more uniform distribution after aging. Furthermore, observation of the morphology of the θ′ phase reveals that the addition of CeLaGdY refines the θ′ phase of the alloy. The results show that the incorporation of CeLaGdY in Al-4Cu-1Mn alloys leads to a reduction in the amount of the T phase and an increase in the volume fraction of the θ′ phase. In addition, the θ′ phase exhibits a finer and denser structure.

### 3.2. Mechanical Properties

**Figure 9:**
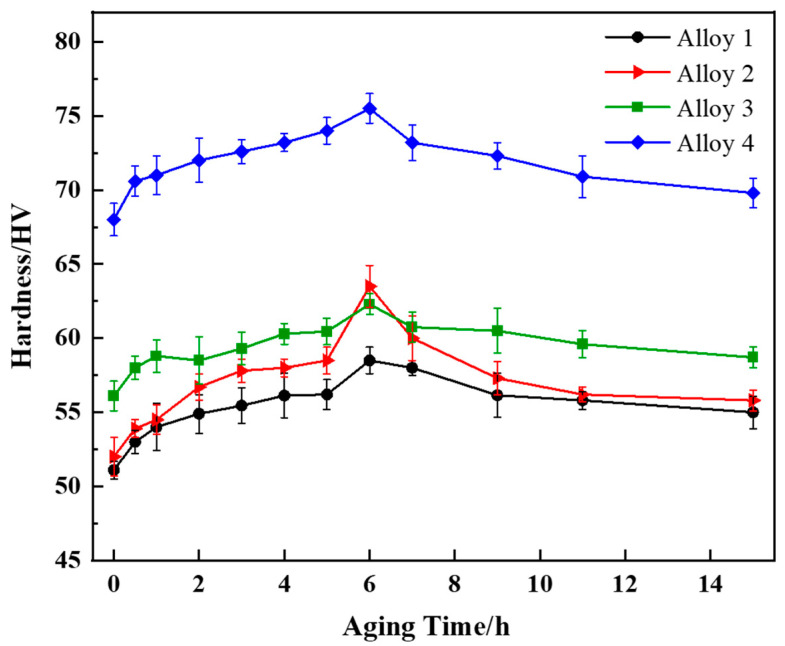
Age-hardening curves of Al-4Cu-1Mn alloys with different kinds of RE.

In general, after appropriate heat treatment, cast alloys can only be used as structural materials [4]. Therefore, alloys with different combinations of RE additions were T6 heat-treated. The alloy was solid solution treated at 535 °C for 16 h and then aged at 180 °C after quenching. Figure 9 shows the aging curves of the studied alloys. The solid solution heat treatment results in Cu and Mn supersaturation. After quenching, the Al solid solution decomposes into T and θ′ phase precipitation and nucleation. Due to the precipitation of strengthening phases, all of the alloys achieved their utmost hardness after being aged at 180 °C for 6 h. Furthermore, it can be seen that the maximum hardnesses of alloy 1, alloy 2, and alloy 3 are 58.5 ± 0.9 HV, 63.5 ± 1.4 HV, and 62.3 ± 0.7 HV, respectively. Notably, alloy 4 with the combined addition of CeLa and GdY has the highest hardness of 76.5 ± 1 HV, which is significantly higher than the other three alloys.

The ultimate tensile strength (UTS), yield strength (YS), and elongation (EL) of various combinations of added RE alloys are shown in Table 2. The unmodified alloy 1 had the lowest values for UTS (190.5 ± 2 MPa) and YS (95.5 ± 4 MPa). The addition of CeLa improves the tensile properties of alloy 2. The addition of GdY increases the tensile strength of alloy 3 but reduces its elongation. The alloy 4 containing CeLa and GdY exhibits superior performance, increasing UTS by 36.9% compared to the alloy without RE and increasing EL by 17.8% compared to the alloy only containing GdY.

**Figure 10:**
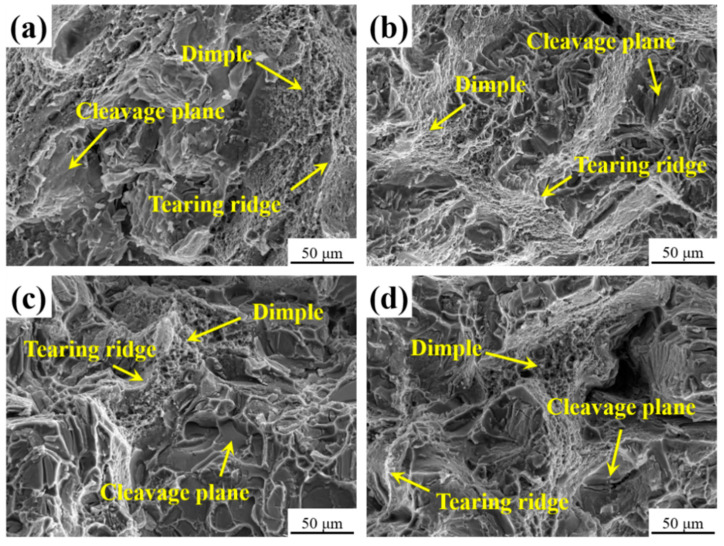
Tensile fracture morphologies of the heat-treated Al-4Cu-1Mn alloys (**a**) Alloy 1, (**b**) Alloy 2 (0.2 CeLa), (**c**) Alloy 3 (0.1 GdY), and (**d**) Alloy 4 (0.2 CeLa + 0.1 GdY).

Figure 10 shows the SEM image of the fracture of the heat-treated tensile specimen. Numerous irregular cleavage planes and holes are distributed at the fracture of the unalloyed RE alloy, as shown in Figure 10a, indicating that the fracture mode of the alloys is mainly brittle fracture. When unmodified, coarse phases in alloy 1 are clearly distributed on the fracture surface, and some cracks appear on the phase surface. These coarse phases act as a source of cracking and greatly reduce the mechanical properties of the alloy. Furthermore, crack initiation and extension predominantly occur along the interface between the coarse phase and the aluminum base. The adjacent cracks are interconnected, leading to material fracture. Figure 10b illustrates a reduction in the quantity of cleavage planes and tearing ridges and an increase in the quantity of dimples in alloy 2, due to the addition of 0.2 wt.% CeLa. When 0.1 wt% GdY is added, the number of dimples in alloy 3 decreases and their depth becomes shallower, as illustrated in Figure 10c. Figure 10d shows that the combined addition of CeLa and GdY leads to an increase in the number of large tough nests and the presence of particles with cracks at the bottom of the tough nests, which are mainly composed of AlCuMnRE. The fracture mode of alloy 4 is a combination of brittle fracture and ductile fracture.

## 4. Discussion

Based on the results presented, the addition of RE can refine the grain, increase the number of intermetallic compounds, and form new RE-rich phases; at the same time, it can change the size and number of the main hardening phase θ′ phase and improve the mechanical properties of the alloys. More specifically, the combined addition of CeLa and GdY provides a better grain refinement effect than the separate addition of CeLa or GdY. The larger radius of Ce (182 pm) and La (187 pm) atoms, compared to Al (118 pm) atoms, leads to greater lattice distortions as Ce and La atoms enter the α(Al) phase, increasing the energy of the whole system. Therefore, CeLa is not effectively dissolved in the α phase but is enriched at grain boundaries, leading to an increase in the concentration gradient at the interfacial front, resulting in compositional supercooling and an increase in nucleation rate. As shown in Formula 1 [40], the critical nucleation radius r* can be reduced by increasing the undercooling required for nucleation ΔT.

(1) $$r*=\frac{2\gamma }{\Delta S}\frac{1}{\Delta T}$$

where γ is the crystal-melt interfacial free energy and ΔS is the melting entropy. Meanwhile, the limited solubility of GdY in the α-phase leads to the aggregation of GdY around the α-phase, which results in a lower liquid-solid surface tension and an increased nucleation rate during solidification. At the same time, GdY is prone to generating intermetallic compounds with other elements that are biased on the grain boundaries, leading to an increase in compositional supercooling near the solid-liquid interface. As a consequence, the above factors work together to further improve grain refinement.

The combined addition of CeLa and GdY resulted in a significant increase in the number of intermetallic compounds in alloy 4 and the formation of a new RE-rich phase. This is due to the limited solubility of GdY in the aluminum matrix, which causes them to solidify at the solid-liquid interface and prevents the diffusion of the alloying elements (Cu and Mn) [22,41]. Simultaneously, RE and alloying elements form AlCuMnRE and AlCuRE phases along the grain boundaries, which interconnect to form a network, and the alloying elements are consumed in large quantities, leading to a reduction in the volume of the Al_2_Cu phase and a change in the microstructure of the alloy.

The combined addition of CeLaGdY resulted in a smaller size and a higher amount of the θ′ phase in the alloy. When CeLa atoms enter the Al matrix, they induce severe lattice distortions, thus increasing system energy. To sustain a low-energy system, more oversaturated vacancies may aggregate around CeLa atoms. Because of their high binding energy for vacancies, CeLa atoms are much easier to migrate and provide the nucleation sites for the formation of the θ′ phase [29]. This leads to an increase in the θ′ phase density. Meanwhile, the addition of GdY reduces the precipitation temperature of the θ′ phase (Figure 5b), enhances its nucleation density, and simultaneously lowers the diffusion rate of the alloying element Cu, leading to a delayed coarsening of the θ′ phase [42].

After heat treatment, a part of the RE dissolves into the Al matrix, filling defects and fully diffusing, eliminating casting defects such as shrinkage holes and microscopic segregation in the alloy, thus improving the mechanical properties of the alloy [43]. The micropores are filled with diffused solute atoms and bonded together. This results in a higher relative density of the alloy and a propensity for the secondary phase to be closely distributed with the matrix grains. Under external stress, the matrix separates from the secondary phase, leading to plastic deformation and the formation of tough nests. When CeLa is added alone, the alloy grain size decreases, and the refined grains with more boundaries hinder crack extension. The grain refinement and strengthening improve the mechanical properties of the alloy [44]. When GdY is added alone, the reticulated AlCuMnGdY phases formed along the grain boundaries in the alloy pin the grain boundaries and form high-density dislocation zones that hinder dislocation movement. Dispersion strengthening enhances the alloy’s strength. Nonetheless, intermetallic compounds will damage the continuity of grain boundaries, and as brittle phases, intermetallic compounds will break when subjected to external forces, thereby reducing the elongation of the alloy [23,45]. The combined addition of CeLa + GdY leads to enhanced grain refinement strengthening and dispersion strengthening, significantly increasing the strength of the alloys while improving their plasticity. Simultaneously, the addition of RE improves the nucleation density of the θ′ phase and retards the coarsening of the θ′ phase. A large amount of finely diffused θ′ phases precipitated from the Al matrix after heat treatment, and the precipitation strengthening effect further improved the mechanical properties of the alloy. For these reasons, the fracture mode of the alloy is altered from brittle fracture partially to ductile fracture.

## 5. Conclusions

In this paper, the effect of the combined addition of CeLa and GdY on the microstructure and mechanical properties of the Al-4Cu-1Mn casting alloy was investigated. Based on the obtained experimental results, the conclusions are as follows:

- The incorporation of 0.2 wt.% CeLa + 0.1 wt.% GdY into the alloy results in sufficient refinement of the α-Al grains, which change from dendrites to equiaxed crystals. The volume of the Al_2_Cu phase diminishes while the amount of the RE-containing phase increases. The average grain size and SADS of α-Al reduce from 1135 µm and 103.75 µm to 372 µm and 42.8 µm, respectively.
- After the combined addition of 0.2 wt% CeLa + 0.1 wt% GdY, the alloy exhibits a bright white AlCuRE phase and a grey AlCuMnRE phase. The secondary phases are closely connected to the matrix grains. The inclusion of RE decreases the precipitation temperature of the θ′ phase, leading the matrix to precipitate numerous finely dispersed θ′ phases. The alloy has excellent overall mechanical properties. The average UTS, YS, and EL of the alloy were measured to be 260.9 MPa, 243.7 MPa, and 1.52%, respectively.

## Figures and Tables

**Table 1:** Normal compositions of the experimental alloys (wt.%).

Alloys	Cu	Mn	CeLa	GdY	Al
Alloy 1	4.0	1.05	0	0	Balance
Alloy 2	4.0	1.05	0.20	0	Balance
Alloy 3	4.0	1.05	0	0.10	Balance
Alloy 4	4.0	1.05	0.20	0.10	Balance

**Table 2:** Results of tensile properties of the heat-treated alloys.

Alloys	UTS (MPa)	YS (MPa)	EL (%)
Alloy 1	190.5 ± 2	95.4 ± 3	3.71 ± 0.5
Alloy 2	215.8 ± 3	104.5 ± 4	4.27 ± 0.6
Alloy 3	240.8 ± 2	206.9 ± 3	1.29 ± 0.3
Alloy 4	260.9 ± 4	243.7 ± 2	1.52 ± 0.2

## Data Availability

The raw/processed data required to reproduce these findings cannot be shared at this time as the data also forms part of an ongoing study.
